# Does Obesity Increase the Risk of Depression in Youth? A Stratified Meta-Analysis of Longitudinal and Cross-Sectional Evidence

**DOI:** 10.3390/bs16050671

**Published:** 2026-04-28

**Authors:** Stănculeț Carmen Ramona, Dan Octavian Rusu, Cristian Delcea

**Affiliations:** 1 Multidisciplinary Doctoral School, Vasile Goldiș Western University of Arad, 310025 Arad, Romania; ghidel.carmen@student.uvvg.ro; 2 Department of Applied Psychology, Babes-Bolyai University of Cluj-Napoca, 4305849 Cluj-Napoca, Romania

**Keywords:** obesity, depression, adolescents, children, body mass index, depressive symptoms, mental health

## Abstract

Obesity and depression in childhood and adolescence represent major public health concerns, yet the nature and direction of their association remain incompletely understood. In the present study, we conducted a systematic review and stratified meta-analysis of epidemiological studies examining the relationship between obesity and depression in youth populations. A total of 945 records were identified through database searches, of which 18 studies met the inclusion criteria for the review. To ensure greater conceptual consistency, quantitative synthesis was restricted to studies examining categorical obesity (typically defined as body mass index (BMI) ≥ 95th percentile) and binary depression outcomes, which formed the longitudinal and cross-sectional meta-analytic cores, analyzed separately. Our longitudinal meta-analysis (k = 4; N = 5581) indicated that obesity was associated with an increased likelihood of subsequent depression (OR = 2.05, 95% CI: 1.40–2.99), whereas the cross-sectional meta-analysis (k = 6; N = 20,278) showed a weaker and non-significant association (OR = 1.40, 95% CI: 0.98–2.00) with moderate heterogeneity. Additional studies that could not be pooled due to differences in exposure or outcome definitions were integrated through narrative synthesis and showed mixed and generally less consistent patterns, broadly supporting the distinction between longitudinal and cross-sectional evidence. Overall, our findings suggest that obesity during childhood and adolescence is associated with an increased likelihood of subsequent depression, while concurrent associations appear more heterogeneous and more difficult to interpret. By distinguishing between study designs and prioritizing comparable effect estimates, this study provides a more transparent synthesis of the current evidence on the relationship between obesity and depression in youth.

## 1. Introduction

The prevalence of obesity among children and adolescents has increased markedly over recent decades and is now widely recognized as one of the most pressing public health challenges affecting younger populations. Global epidemiological estimates indicate that the number of children and adolescents living with obesity has risen dramatically since the late twentieth century, reflecting large-scale changes in diet, physical activity patterns, and broader social environments (Ng et al., 2014; NCD Risk Factor Collaboration, 2017). Recent global estimates indicate that over 390 million children and adolescents aged 5–19 years are overweight, including approximately 160 million living with obesity (World Health Organization, 2025). Although the epidemic initially emerged in high-income societies, recent evidence indicates that childhood obesity is now expanding rapidly in many developing regions, suggesting that excess body weight in youth has become a global phenomenon (Ng et al., 2014; NCD Risk Factor Collaboration, 2017).

The consequences of obesity during childhood and adolescence extend well beyond physical health. Excess adiposity during development has been associated with an increased risk of cardiometabolic conditions such as insulin resistance, hypertension, and dyslipidemia (Reilly & Kelly, 2011). At the same time, a growing body of studies suggests that obesity in youth may also be linked to psychosocial difficulties, including lower self-esteem, poorer perceived health, and reduced health-related quality of life (Griffiths et al., 2010; Swallen et al., 2005). Adolescence represents a developmental stage characterized by heightened sensitivity to social evaluation and body image concerns, which may amplify the psychological consequences of excess body weight (Puhl & Heuer, 2009; Griffiths et al., 2010). Experiences such as peer victimization, social marginalization, and stigma related to body weight may further contribute to emotional distress among adolescents with obesity (Puhl & Heuer, 2009).

In epidemiological research, obesity in youth is typically operationalized using body mass index (BMI), adjusted for age and sex. BMI is widely used in population-based studies because it provides a practical proxy indicator of adiposity that allows comparisons across large samples. Most pediatric epidemiological studies define obesity as BMI values at or above the 95th percentile for age and sex according to standardized growth references, such as those developed by the Centers for Disease Control and Prevention or the World Health Organization (Cole et al., 2000; Kuczmarski et al., 2002). Normal weight is generally defined as BMI values below the 85th percentile, while intermediate percentiles correspond to overweight status (Cole et al., 2000). Although BMI does not directly measure body fat, it remains the most widely used metric for identifying obesity in large-scale epidemiological investigations involving children and adolescents (Reilly & Kelly, 2011).

Parallel to the rising prevalence of obesity, depression among adolescents has also emerged as a major public health concern. Major depressive disorder represents one of the most common psychiatric conditions during adolescence and is associated with substantial impairment in emotional, social, and academic functioning (Thapar et al., 2012). Epidemiological evidence suggests that depressive symptoms and mood disorders among adolescents have increased in several countries over the past two decades (Mojtabai et al., 2016; Twenge et al., 2019). Because depressive disorders often first emerge during adolescence and may persist into adulthood, understanding the potential risk factors contributing to their development has become an important focus of both clinical and population health research (Thapar et al., 2012).

Depression in epidemiological studies is typically assessed using either structured diagnostic interviews or validated symptom scales. Diagnostic instruments commonly employed in large cohort studies include the Diagnostic Interview Schedule for Children (DISC), the Structured Clinical Interview for DSM Disorders (SCID), and the Kiddie Schedule for Affective Disorders and Schizophrenia (K-SADS), which allow researchers to establish psychiatric diagnoses according to standardized diagnostic criteria (Kaufman et al., 1997; Shaffer et al., 2000). Alternatively, many population-based studies assess depressive symptoms using validated self-reporting instruments that capture symptom severity along a continuous dimension. Widely used examples include the Center for Epidemiologic Studies Depression Scale (CES-D), the Patient Health Questionnaire (PHQ-9), and the Child Depression Inventory (CDI), all of which have demonstrated strong psychometric properties in both clinical and community samples (Radloff, 1977; Kovacs, 1985; Kroenke et al., 2001).

Given the increasing prevalence of both obesity and depression during adolescence, a substantial body of research has examined whether these conditions may be linked. Several theoretical mechanisms have been proposed to explain the potential associations between obesity and depressive outcomes. Psychosocial pathways have received considerable attention, particularly those related to weight stigma, teasing, and discrimination associated with body weight (Puhl & Heuer, 2009). Adolescents with obesity may experience social exclusion or negative peer interactions, which may contribute to body dissatisfaction, reduced self-worth, and depressive symptoms (Griffiths et al., 2010). At the same time, biological pathways have also been proposed, including inflammatory processes and metabolic dysregulation associated with adiposity that may influence neurobiological systems involved in mood regulation (Milaneschi et al., 2019). Furthermore, obesity and depression share several behavioral risk factors, including reduced physical activity, sleep disturbances, and unhealthy dietary behaviors, suggesting that both conditions may be embedded within broader lifestyle and environmental contexts (Chaput et al., 2016; Milaneschi et al., 2019).

Despite these plausible mechanisms, empirical findings examining the association between obesity and depression in youth remain mixed. Some studies report positive associations between excess body weight and depressive outcomes, whereas others have found weak or non-significant relationships. For example, Swallen et al. (2005) observed that overweight and obese adolescents reported poorer health-related quality of life, although associations with depressive outcomes were not consistently strong across analyses. Similarly, Wardle et al. (2006) found only modest differences in emotional symptoms across weight categories. In contrast, other studies have reported more robust associations between obesity and depressive outcomes in youth populations (Bazargan-Hejazi et al., 2010; Smith & Mason, 2022). However, many of these studies rely on cross-sectional designs, which limit the ability to determine whether obesity precedes depression or whether depressive symptoms contribute to weight gain. Longitudinal research has attempted to clarify the temporal relationship between these conditions. For example, Anderson et al. (2007) reported that obesity during adolescence was associated with increased risk of subsequent mood disorders in a community cohort followed into adulthood. Similarly, Roberts and Duong (2013) observed that obesity predicted later depressive disorders in adolescents in a prospective cohort study. Previous meta-analytic syntheses have also attempted to integrate evidence across studies. Luppino et al. (2010) reported that obesity was associated with an increased risk of subsequent depression, while Mannan et al. (2016) found modest prospective associations between obesity and depressive outcomes across different age groups.

Nevertheless, several methodological challenges continue to be present in the existing literature. First, a large proportion of studies examining obesity and depression rely on cross-sectional data, which does not allow conclusions regarding temporal ordering or causality (Luppino et al., 2010). Second, substantial heterogeneity exists in how both obesity and depression are measured across studies, including differences in exposure definitions, outcome instruments, and analytic approaches (Mannan et al., 2016). Third, many epidemiological studies do not report effect sizes in formats that can be readily incorporated into quantitative synthesis. Finally, several previous syntheses have combined cross-sectional and longitudinal evidence within the same pooled analyses, potentially obscuring important differences in study design and interpretation (Luppino et al., 2010; Mannan et al., 2016).

As a result, the temporal relationship between obesity and depression during adolescence remains incompletely understood. The current evidence does not clearly distinguish whether obesity increases the risk of developing depression, whether depressive symptoms contribute to weight gain, or whether both conditions reflect shared underlying determinants.

To address these limitations, we conducted a systematic review and stratified meta-analysis of epidemiological studies examining the association between obesity and depression in youth using a structured analytical framework. Rather than pooling all available studies indiscriminately, prospective longitudinal studies examining whether baseline obesity predicts subsequent depression were prioritized as the primary analytic core. To ensure the comparability of effect sizes, quantitative pooling was restricted to longitudinal studies reporting categorical definitions of obesity (typically defined as BMI ≥ 95th percentile) and binary depression outcomes, allowing for the calculation of odds ratios. Additional longitudinal evidence that could not be quantitatively synthesized due to differences in exposure definitions or outcome measures was incorporated through narrative synthesis. Cross-sectional studies examining the concurrent association between obesity and depression were analyzed separately in a secondary meta-analysis.

By distinguishing evidence according to study design and the comparability of effect size estimates, our stratified meta-analytic approach provides a clearer and more transparent synthesis of research examining the relationship between obesity and depression during adolescence. Through this framework, we aim to contribute to the literature by prioritizing longitudinal evidence, explicitly separating findings by study design (longitudinal vs. cross-sectional), and integrating quantitative and narrative evidence to better characterize the complex relationship between obesity and depression in youth.

Accordingly, we address the following research question in our study:

*To what extent is obesity associated with depression in children and adolescents, and how does this association vary across study designs and methodological approaches?*

In particular, we examine whether obesity predicts subsequent depression in longitudinal studies and whether similar patterns are observed in cross-sectional investigations of concurrent associations.

### The Present Study

In the present study, we aimed to systematically examine the association between obesity and depression in children and adolescents through a structured synthesis of epidemiological evidence. Given the substantial methodological variability across studies, including differences in study design and measurement approaches, our analysis was designed to distinguish between longitudinal and cross-sectional evidence in order to better clarify the nature of this association.

Specifically, we investigated whether obesity is associated with subsequent depression in longitudinal studies and whether similar patterns are observed in cross-sectional research examining concurrent associations. By integrating quantitative meta-analysis with a structured narrative synthesis of additional evidence, we seek to provide a more transparent and conceptually consistent understanding of the relationship between obesity and depression in youth. Accordingly, we address the following research question: To what extent is obesity associated with depression in children and adolescents, and how does the nature of this association vary as a function of the study design (longitudinal vs. cross-sectional)?

## 2. Methods

### 2.1. Study Design and Analytical Framework

In the present study, we employed a systematic review and meta-analytic design to synthesize epidemiological evidence examining the association between obesity and depression in children and adolescents. Our review employed a stratified framework distinguishing between a core meta-analytic sample and an extended body of evidence included for narrative synthesis. The study was conducted in accordance with the Preferred Reporting Items for Systematic Reviews and Meta-Analyses (PRISMA) 2020 guidelines, ensuring transparent identification, screening, and inclusion of relevant studies. Although no formal pre-registered protocol (e.g., PROSPERO) was prepared for this review, the study design, eligibility criteria, and analytical framework were defined a priori and followed PRISMA 2020 guidelines to ensure methodological transparency.

Meta-analysis represents a statistical framework that allows effect sizes from independent studies to be quantitatively integrated in order to estimate an overall association while accounting for sampling variability and between-study heterogeneity (Borenstein et al., 2009; Higgins et al., 2019). Compared with narrative summaries of the literature, meta-analysis offers a more rigorous method for evaluating both the magnitude and consistency of reported associations across epidemiological investigations.

Because studies examining the relationship between obesity and depression in youth vary substantially in design and measurement approaches, we adopted a stratified analytic framework in the present synthesis. The methodological literature has emphasized that effect sizes derived from cross-sectional and longitudinal studies address different research questions and should not be indiscriminately pooled within a single quantitative estimate (Borenstein et al., 2009; Higgins et al., 2019). Cross-sectional studies capture concurrent associations between variables, whereas longitudinal studies provide evidence relevant to temporal ordering and potential causal interpretation.

In accordance with these considerations, prospective longitudinal studies examining whether obesity predicts subsequent depression were treated as the primary analytic core of the meta-analysis. Cross-sectional studies examining concurrent associations between obesity and depressive outcomes were analyzed separately in a secondary quantitative synthesis. Additional studies that did not report effect sizes in formats suitable for pooling or those that used exposure or outcome definitions that were not directly comparable were incorporated through narrative synthesis in order to contextualize the quantitative findings.

### 2.2. Literature Search Strategy

A structured literature search was conducted between 3 February and 27 February 2026 to identify epidemiological studies examining the association between obesity and depression in children and adolescents. Structured search procedures are commonly used in meta-analytic research to improve transparency and reduce the likelihood of selective study inclusion (Higgins et al., 2019; Page et al., 2021).

The search was performed across four major bibliographic databases widely used in health and behavioral sciences research: PubMed, PsycINFO, Embase, and the Web of Science Core Collection. These databases provide extensive coverage of biomedical, psychological, and interdisciplinary research, thereby increasing the likelihood of identifying relevant epidemiological studies (Higgins et al., 2019). A supplementary search was also conducted using Google Scholar to identify potentially relevant studies that might not have been captured through database indexing.

Our search strategy combined controlled vocabulary terms (e.g., MeSH terms in PubMed and Emtree terms in Embase) with free-text keywords related to obesity, depression, and youth populations. Search terms were adapted for each database to reflect differences in indexing systems and search functionalities.

The full database-specific search strategies, including Boolean operators, controlled vocabulary terms, and field specifications, are provided in Supplementary Table S1 to ensure full reproducibility of the search process.

No restrictions were applied regarding publication year, though only studies published in English and conducted in human populations were considered eligible for inclusion.

The initial search identified 945 records. After the removal of 16 duplicate entries, 929 unique articles remained for screening.

### 2.3. Study Selection Process

Study selection was conducted independently by two reviewers (S.C.R. and D.O.R.) in accordance with recommended practices for systematic reviews to reduce selection bias (Higgins et al., 2019; Page et al., 2021). Titles and abstracts were initially screened to identify studies potentially relevant to the research question.

Full-text articles were subsequently assessed for eligibility based on the predefined inclusion and exclusion criteria. Discrepancies between reviewers were resolved through discussion and consensus. In cases where consensus could not be reached, a third reviewer (C.D.) was consulted to resolve disagreements.

In addition to database screening, backward citation tracking of relevant reviews was performed to identify additional eligible studies (Greenhalgh & Peacock, 2005).

**Figure 1:**
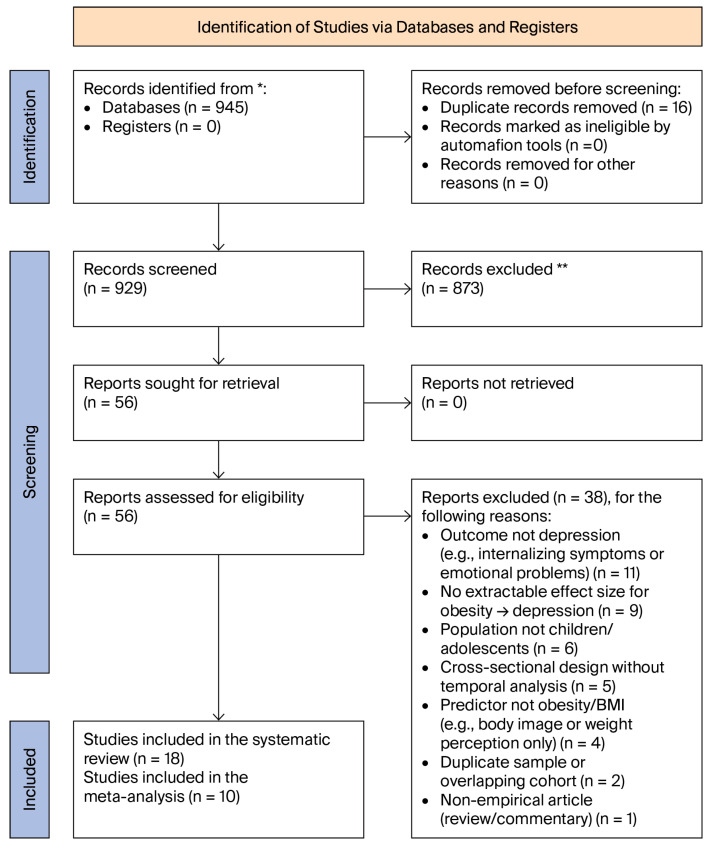
PRISMA 2020 flow diagram illustrating the identification, screening, eligibility assessment, and inclusion of studies in the present systematic review and meta-analysis on obesity and depression in children and adolescents. * the number of records identified from each database or register. ** indicate how many records were excluded.

The study selection process is illustrated in Figure 1.

### 2.4. Eligibility Criteria

Studies were considered eligible for inclusion in the quantitative meta-analysis if they examined the association between obesity and depression among children or adolescents and reported effect sizes, allowing for the calculation of odds ratios or an equivalent association measure. Obesity needed to be operationalized using BMI or BMI-based categories derived from standardized growth references, which are widely used in pediatric epidemiological research, such as those developed by the Centers for Disease Control and Prevention and the International Obesity Task Force (Cole et al., 2000; Kuczmarski et al., 2002). Depression outcomes were required to be measured using either diagnostic interviews based on established psychiatric criteria or validated symptom scales commonly used in epidemiological research.

To ensure the comparability of effect sizes across studies, inclusion in the quantitative meta-analysis was restricted to studies reporting categorical definitions of obesity (typically defined as BMI ≥ 95th percentile adjusted for age and sex) and binary depression outcomes, allowing for the calculation of odds ratios. This restriction was an intentional methodological choice to prioritize conceptual coherence and the validity of pooled estimates by avoiding the aggregation of non-equivalent constructs (e.g., continuous symptom scores versus clinical diagnoses), which would require additional transformation assumptions and could compromise interpretability, even at the expense of reducing the number of eligible studies. Studies were excluded from the quantitative meta-analysis if the statistical information required to compute effect sizes was not available. However, such studies were retained for qualitative integration where relevant, allowing a broader evidence base to be considered in the interpretation of findings.

No restrictions were applied regarding publication date, though only studies published in English were considered eligible for inclusion.

### 2.5. Data Extraction

Data extraction was performed independently by two reviewers using a structured extraction template developed for the purposes of the present meta-analysis. Standardized extraction procedures are recommended in meta-analytic research to improve reliability and ensure consistent recording of study characteristics and effect sizes (Borenstein et al., 2009; Higgins et al., 2019).

For each study, information was extracted regarding the author and year of publication, country of study, study design, sample size, participant age range, operational definition of obesity, definition of the comparison group, depression measurement instrument, outcome type, and reported association estimates. When multiple models were reported, preference was given to effect sizes derived from analyses adjusted for relevant covariates, as adjusted estimates may provide more accurate representations of associations by accounting for potential confounding variables (Greenland et al., 1999).

### 2.6. Statistical Analysis

All statistical analyses were conducted using R version 4.3.2 (R Foundation for Statistical Computing, Vienna, Austria). Meta-analyses were performed using the ‘meta’ package (version 6.5-0), applying random-effects models to account for between-study variability, which assume that true effect sizes may vary across studies due to differences in populations, measurement approaches, and study contexts (Borenstein et al., 2009). Random-effects models are therefore generally recommended when synthesizing epidemiological evidence where heterogeneity between studies is expected.

Effect sizes were standardized as odds ratios (ORs) with corresponding 95% confidence intervals. Odds ratios represent a common measure of association in epidemiological studies examining conditions with binary outcomes, such as depressive disorders.

Between-study heterogeneity was evaluated using several complementary statistics. Cochran’s Q statistic was used to test whether the observed variability in effect sizes exceeded that expected by sampling error alone. The I^2^ statistic was used to quantify the proportion of total variance attributable to between-study heterogeneity rather than chance, while the between-study variance parameter τ^2^ provided an estimate of the dispersion of true effect sizes (Higgins et al., 2003, 2019).

Sensitivity analyses were conducted to examine the robustness of pooled estimates. Specifically, leave-one-out analyses were performed in which each study was sequentially removed from the meta-analysis to evaluate the influence of individual studies on the pooled effect size. Sensitivity analyses are commonly recommended in meta-analytic research to assess the stability of overall estimates and identify potential outliers (Borenstein et al., 2009).

Potential small-study effects were evaluated using regression-based tests for funnel plot asymmetry. Because odds ratios were used as the primary effect-size metric, Peters’ test was used as the primary test for small-study effects in the cross-sectional meta-analysis, while Egger’s regression test was performed as an exploratory analysis. In the longitudinal meta-analysis, Egger’s regression test was also treated as exploratory because of the small number of included studies. Given the limited number of studies in both meta-analyses, all tests for small-study effects should be interpreted with caution (Peters et al., 2006).

### 2.7. Risk-of-Bias and Certainty of Evidence Assessment

The risk of bias of the included studies was evaluated using a domain-based assessment framework appropriate for observational studies of exposure. This approach was adopted to provide a more detailed and transparent evaluation of potential biases compared to summary quality scales.

Studies were assessed across the following domains: selection of participants, comparability of study groups (including control for confounding), measurement of exposure (obesity), measurement of outcomes (depression), and adequacy of follow-up in longitudinal studies. Each domain was rated as having a low, moderate, or high risk of bias based on predefined criteria.

Risk-of-bias assessments were conducted independently by two reviewers (S.C.R. and D.O.R.), with discrepancies resolved through discussion and consensus, and consultation with a third reviewer (C.D.) when necessary.

To complement the risk-of-bias assessment, the overall certainty of evidence was evaluated using the GRADE approach. Certainty ratings were determined separately for longitudinal and cross-sectional evidence, considering risk of bias, inconsistency, indirectness, imprecision, and potential publication bias.

While the Newcastle–Ottawa Scale (NOS) was retained as a supplementary descriptive tool to ensure comparability with prior meta-analyses, the interpretation of study quality was based primarily on the domain-level assessment and GRADE framework.

## 3. Results

### 3.1. Study Selection Findings

The literature search identified 945 records across the electronic databases searched. After removing 16 duplicate entries, 929 unique records remained for screening. Titles and abstracts were evaluated to determine whether the studies examined the association between obesity and depression among children or adolescents. Articles that appeared potentially relevant were subsequently assessed through full-text review.

Studies were retained if they examined the association between obesity and depression among youth populations and reported effect-size estimates, allowing for quantitative meta-analysis. Studies that did not report extractable effect sizes or those that used exposure or outcome definitions not directly comparable with the analytical framework were retained for narrative synthesis. In total, 18 studies met the inclusion criteria for the systematic review and were organized according to the stratified analytical framework described in Section 2.1 of Section 2.

### 3.2. Study Characteristics

The studies included in the systematic review comprised both longitudinal and cross-sectional designs, including eight longitudinal cohort studies and 10 cross-sectional population-based studies. The characteristics of the included studies are presented in Table 1.

Although the studies included in the review were conducted across diverse geographic regions, most were conducted in the United States, which may limit the generalizability of the findings to other populations. Across studies, obesity was operationalized using BMI thresholds adjusted for age and sex, most commonly defined as BMI values at or above the 95th percentile according to established growth references such as those developed by the Centers for Disease Control and Prevention and the International Obesity Task Force.

Depression outcomes were assessed using structured diagnostic interviews or validated symptom scales commonly used in epidemiological research. Diagnostic instruments included structured interviews based on DSM criteria, such as the Diagnostic Interview Schedule for Children and the Kiddie Schedule for Affective Disorders and Schizophrenia, which were used in several longitudinal and cross-sectional investigations (Anderson et al., 2007; Boutelle et al., 2010; Marmorstein et al., 2014; Merikangas et al., 2012; Roberts & Duong, 2013). Other studies assessed depressive symptoms using validated self-reporting instruments, including the Center for Epidemiologic Studies Depression Scale, the Child Depression Inventory, and related scales (Needham & Crosnoe, 2005; Özmen et al., 2007; Swallen et al., 2005; Wardle et al., 2006).

The stratified analytical framework distinguished between prospective longitudinal studies examining whether obesity predicted subsequent depression and cross-sectional studies examining concurrent associations between obesity and depressive outcomes. Four studies met the criteria for inclusion in the longitudinal meta-analysis (Anderson et al., 2007; Boutelle et al., 2010; Marmorstein et al., 2014; Roberts & Duong, 2013), and six studies met the criteria for inclusion in the cross-sectional meta-analysis (Bazargan-Hejazi et al., 2010; Delgado-Floody et al., 2020; Merikangas et al., 2012; Sjöberg et al., 2005; Smith & Mason, 2022; Swallen et al., 2005).

Additional studies were incorporated as extended evidence because their exposure definitions, outcome measures, or statistical reporting differed from those used by the studies included in the meta-analysis. These included longitudinal studies examining associations between continuous BMI measures and depressive symptoms (Colozza et al., 2025; Hammerton et al., 2014; Pryor et al., 2016; Wang et al., 2014), as well as cross-sectional investigations that assessed depressive or emotional symptoms using continuous scales or broader emotional constructs (Needham & Crosnoe, 2005; Özmen et al., 2007; Wardle et al., 2006).

### 3.3. Meta-Analysis of Longitudinal Studies

The primary meta-analysis focused on prospective cohort studies examining whether obesity during childhood or adolescence predicted the subsequent onset of depression. Four longitudinal studies met the criteria for inclusion in this primary meta-analysis (Anderson et al., 2007; Boutelle et al., 2010; Marmorstein et al., 2014; Roberts & Duong, 2013). As shown in Table 2, across these studies, obesity was operationalized as a BMI value at or above the 95th percentile, adjusted for age and sex. Depression outcomes were assessed using structured diagnostic interviews based on DSM criteria, allowing for the identification of major depressive disorder as a binary outcome. Effect sizes were standardized as odds ratios. When a study reported subgroup-specific estimates, such as the sex-stratified effects reported in Anderson et al. (2007), these estimates were combined within the study to produce a single study-level effect prior to inclusion in the meta-analysis. Random-effects models were used to synthesize the prospective association between obesity and subsequent depression (see Table 2).

The random-effects meta-analysis yielded a pooled effect estimate of OR = 2.05 (95% CI: 1.40–2.99), indicating that obesity was associated with an increased likelihood of subsequent depression. Statistical heterogeneity was negligible (Q = 1.22; I^2^ = 0%; τ^2^ = 0.00), although the confidence interval around I^2^ was wide, reflecting uncertainty due to the small number of included studies.

**Figure 2:**
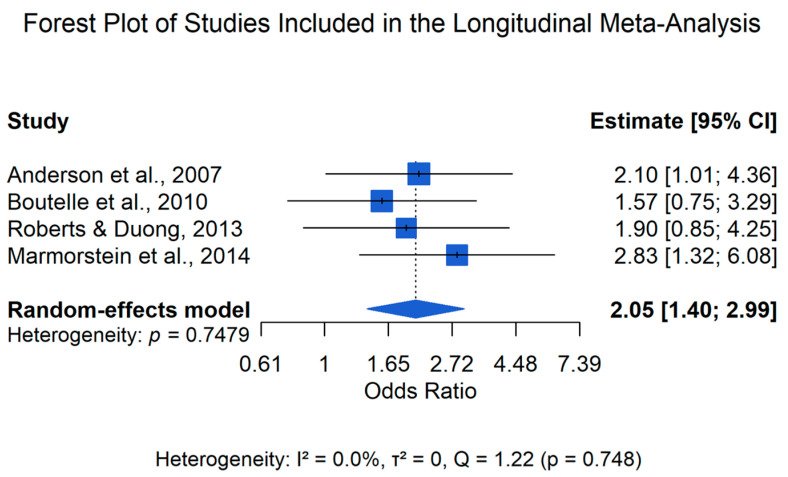
Forest plot of the longitudinal meta-analysis examining whether adolescent obesity predicts subsequent depression. Effect sizes are presented as odds ratios with 95% confidence intervals, and the pooled estimate was calculated using a random-effects model. Studies included: Anderson et al. (2007), Boutelle et al. (2010), Roberts and Duong (2013), and Marmorstein et al. (2014). Statistical heterogeneity across the four longitudinal studies was negligible (Q = 1.89, I^2^ = 0.0%, τ^2^ = 0.00). However, given the small number of studies included, this absence of detected heterogeneity should be interpreted with caution. The low heterogeneity may partly reflect the strict inclusion criteria applied in the longitudinal core analysis, which restricted the sample to studies using comparable definitions of categorical obesity and binary depression outcomes.

The individual study estimates and pooled effect are presented in Figure 2.

Sensitivity analyses were conducted using a leave-one-out approach in which each study was sequentially removed and the pooled estimate recalculated. Across these analyses, the pooled odds ratios ranged from 2.09 to 2.61, and the association remained statistically significant in all iterations, indicating that the pooled estimate was not driven by any single study.

### 3.4. Extended Longitudinal Evidence

Four additional longitudinal studies examined the associations between body weight and depressive outcomes but could not be included in the meta-analysis because their exposure definitions or outcome metrics were not directly compatible with the analytical framework of categorical obesity and binary outcomes for depression (Colozza et al., 2025; Hammerton et al., 2014; Pryor et al., 2016; Wang et al., 2014).

These studies differed from the studies included in the meta-analysis primarily in their operationalization of exposure or outcome variables. In several cases, body weight was analyzed using continuous BMI measures rather than categorical obesity thresholds. Hammerton et al. (2014), for example, examined prospective associations between standardized BMI and depressive outcomes using data from the Early Prediction of Adolescent Depression (EPAD) cohort and the Avon Longitudinal Study of Parents and Children (ALSPAC). In these analyses, BMI was modeled as a continuous predictor, and depressive outcomes included both diagnostic and symptom-based measures.

Similarly, Wang et al. (2014) analyzed the association between BMI z-scores and depressive symptoms measured using the Patient Health Questionnaire-9 among adolescents in Hong Kong. In this study, both exposure and outcome variables were analyzed as continuous measures rather than categorical clinical outcomes.

Other longitudinal studies examined the developmental trajectories of weight status across childhood and adolescence, rather than categorical obesity measured at a single time point. Pryor et al. (2016) investigated the trajectories of overweight status and their relationship with depressive symptoms measured during adolescence using the Child Depression Inventory. In this study, overweight and obesity were analyzed together as a combined exposure category, and depressive symptoms were assessed as continuous outcomes. The longitudinal study by Colozza et al. (2025) used data from the Indonesia Family Life Survey and analyzed the relationship between overweight status and depressive symptoms, measured using the CES-D-10 scale, across adolescence and adulthood.

Despite these methodological differences, the findings of the extended longitudinal studies were broadly consistent with the direction of effects observed in the core meta-analysis. Several studies reported positive associations between higher body weight and subsequent depressive outcomes, although effect sizes varied and were often attenuated when continuous measures were used. For example, while Hammerton et al. (2014) and Wang et al. (2014) found modest associations between BMI and depressive symptoms when modeled continuously, Pryor et al. (2016) reported associations between overweight trajectories and internalizing symptoms, suggesting that developmental patterns of weight status may be relevant for later emotional outcomes. Similarly, Colozza et al. (2025) observed associations between overweight status and depressive symptoms across adolescence and adulthood. Overall, these findings provide supplementary evidence supporting a potential association between body weight and depressive outcomes, although differences in measurement and study design limit direct comparability with the core meta-analytic results.

### 3.5. Meta-Analysis of Cross-Sectional Studies

A separate meta-analysis was conducted for studies employing a cross-sectional design that examined the concurrent association between obesity and depression. Six studies met the criteria for inclusion in the cross-sectional meta-analysis (Bazargan-Hejazi et al., 2010; Delgado-Floody et al., 2020; Merikangas et al., 2012; Sjöberg et al., 2005; Smith & Mason, 2022; Swallen et al., 2005).

As shown in Table 3, across these studies, obesity was operationalized using categorical BMI thresholds consistent with epidemiological definitions of obesity, and depression outcomes were assessed using either structured diagnostic interviews or validated screening instruments that allowed for the classification of depression as a binary outcome. Effect sizes were standardized as odds ratios and synthesized using a random-effects model (see Table 3).

The effect estimates represent odds ratios comparing adolescents with obesity to normal-weight reference groups.

The random-effects meta-analysis yielded a pooled effect estimate of OR = 1.40 (95% CI: 0.98–2.00) with moderate statistical heterogeneity (Q = 12.71; I^2^ = 60.65%; τ^2^ = 0.119).

The pooled effect estimate indicated a positive but statistically non-significant association between obesity and depression, with a pooled odds ratio of 1.40 and a 95% confidence interval ranging from 0.98 to 2.00.

**Figure 3:**
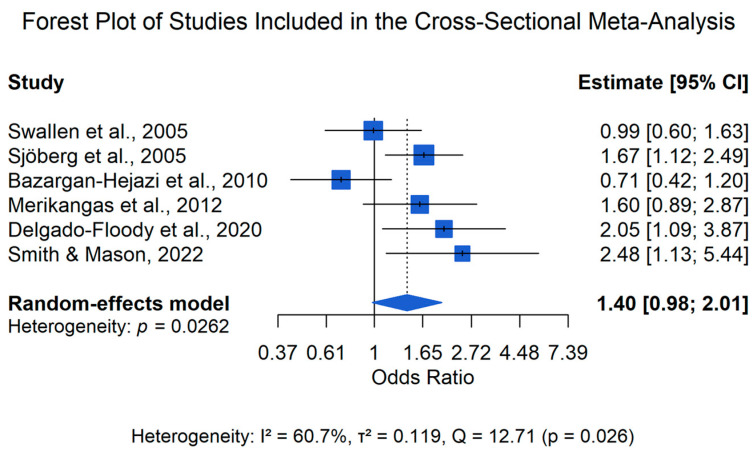
Forest plot of the cross-sectional meta-analysis examining the concurrent association between obesity and depression in adolescents. Effect sizes are presented as odds ratios with 95% confidence intervals, and the pooled estimate was calculated using a random-effects model. Studies included: Swallen et al. (2005), Sjöberg et al. (2005), Bazargan-Hejazi et al. (2010), Merikangas et al. (2012), Delgado-Floody et al. (2020), and Smith and Mason (2022). Statistical heterogeneity across the cross-sectional studies was moderate (Q = 12.71, I^2^ = 60.65%, τ^2^ = 0.119), likely reflecting variability in study design, measurement approaches, and population characteristics.

The individual study estimates and pooled effect for the cross-sectional meta-analysis are presented in Figure 3.

As shown in Table 4, sensitivity analyses using a leave-one-out procedure indicated that the pooled odds ratios varied between 1.29 and 1.59 depending on which study was removed from the analysis. The removal of the study conducted by Bazargan-Hejazi et al. (2010) resulted in the largest change in the pooled estimate and substantially reduced heterogeneity.

### 3.6. Extended Cross-Sectional Evidence

Three additional cross-sectional studies were identified that examined the associations between body weight and depressive or emotional symptoms but were not included in the meta-analysis because their exposure or outcome definitions differed from those of the studies included in the meta-analysis (Needham & Crosnoe, 2005; Özmen et al., 2007; Wardle et al., 2006).

In several of these studies, body weight was analyzed using continuous BMI measures rather than categorical obesity thresholds. Needham and Crosnoe (2005), for example, analyzed associations between BMI and depressive symptoms using data from the National Longitudinal Study of Adolescent Health. In this study, BMI was treated as a continuous predictor, and depressive symptoms were measured using the Center for Epidemiologic Studies Depression Scale as a continuous outcome.

Other studies examined depressive symptoms using continuous scales or broader emotional constructs. Özmen et al. (2007) investigated the relationship between obesity and depressive symptoms among Turkish adolescents using the Children’s Depression Inventory but did not report effect-size estimates in a format that allowed for their inclusion in the meta-analysis.

Similarly, Wardle et al. (2006) examined associations between obesity and emotional symptoms among British adolescents using data from the HABITS cohort. Emotional difficulties were assessed using the Strengths and Difficulties Questionnaire emotional symptoms subscale, which captures broader emotional problems rather than depressive disorders specifically.

### 3.7. Publication Bias

Potential small-study effects were evaluated using regression-based tests for funnel plot asymmetry. Because odds ratios were used as the primary effect-size metric, Peters’ test was used as the primary test for the cross-sectional meta-analysis, while Egger’s regression test was performed as an exploratory analysis. For the longitudinal meta-analysis, Egger’s regression test was again used due to the small number of studies included.

**Figure 4:**
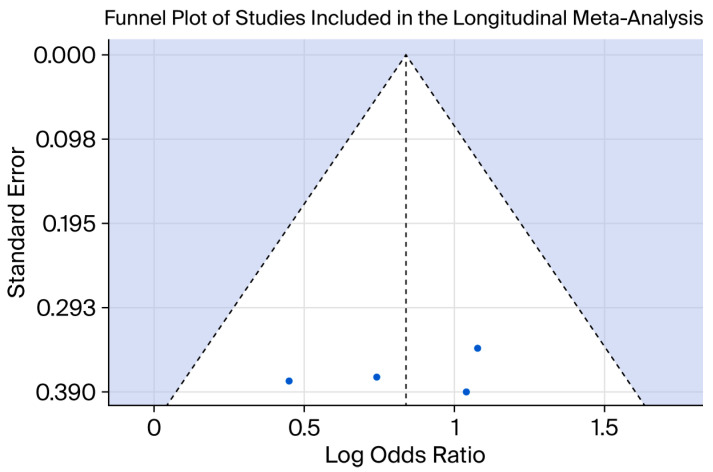
Funnel plot assessing potential small-study effects in the longitudinal meta-analysis examining the association between adolescent obesity and subsequent depression. Each point represents an individual study. The vertical dashed line indicates the pooled effect estimate, and the diagonal dashed lines represent the expected 95% confidence region around the summary effect. The shaded area illustrates the region of expected symmetry in the absence of publication bias. For the cross-sectional meta-analysis, Peters’ regression test did not indicate statistically significant evidence of small-study effects. Egger’s regression test, performed as an exploratory analysis, likewise did not suggest significant funnel plot asymmetry. The funnel plot for the cross-sectional meta-analysis is presented in Figure 5.

Egger’s regression test did not indicate statistically significant funnel plot asymmetry in the longitudinal meta-analysis. A visual representation of the funnel plot is presented in Figure 4.

**Figure 5:**
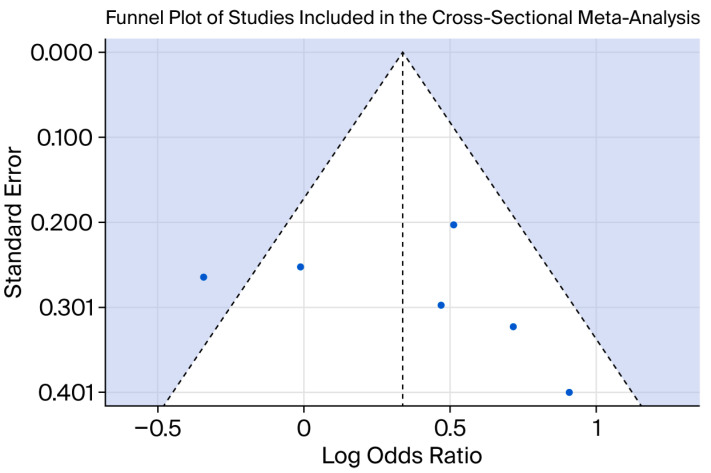
Funnel plot assessing potential small-study effects in the cross-sectional meta-analysis examining the concurrent association between obesity and depression in adolescents. Each point represents an individual study. The vertical dashed line indicates the pooled effect estimate, and the diagonal dashed lines represent the expected 95% confidence region around the summary effect. The shaded area illustrates the region of expected symmetry in the absence of publication bias. Results of the publication bias tests are summarized in Table 5.

**Table 5:** Tests for small-study effects in the longitudinal and cross-sectional meta-analyses.

Analysis	Test	*p*-Value
Longitudinal meta-analysis	Egger’s regression test	0.596
Cross-sectional meta-analysis	Peters’ test	0.536
Cross-sectional meta-analysis	Egger’s regression test	0.554

Note: Small-study effects were evaluated using regression-based tests for funnel plot asymmetry. Peters’ test was used as the primary test for the cross-sectional meta-analysis because odds ratios were used as the effect-size metric. Egger’s regression test was also performed as an exploratory analysis. Given the limited number of studies included in the meta-analyses, these tests should be interpreted cautiously.

Overall, the statistical tests did not indicate statistically significant evidence of small-study effects in either the longitudinal or cross-sectional meta-analysis. However, these tests are underpowered given the small number of included studies and should therefore be interpreted with caution. Consequently, the absence of statistically significant findings should not be interpreted as evidence of the absence of publication bias.

### 3.8. Risk-of-Bias Assessment

The risk-of-bias assessment indicated a variability in methodological quality across the included studies when evaluated across key domains. Longitudinal studies generally demonstrated a lower risk of bias, particularly in the measurement of exposure and outcomes and in the temporal ordering of variables. However, some studies showed limitations in the control of confounding variables, which may influence the observed associations.

In contrast, cross-sectional studies exhibited a greater variability in risk of bias across domains. In particular, a higher risk of bias was observed in the domains of comparability (due to limited adjustment for potential confounders) and participant selection. Additionally, the absence of temporal information in cross-sectional designs represents an inherent limitation, restricting our ability to draw conclusions regarding the direction of the association between obesity and depression (Higgins et al., 2019).

Overall, these findings suggest that the longitudinal evidence is characterized by a relatively lower risk of bias, whereas cross-sectional evidence should be interpreted more cautiously due to the greater methodological variability across studies. These patterns are consistent with the known methodological differences between longitudinal and cross-sectional designs in observational research.

The results of the risk-of-bias assessment are presented in detail in Table 6.

### 3.9. Certainty of Evidence

The overall certainty of evidence was evaluated using the GRADE approach, considering risk of bias, inconsistency, indirectness, imprecision, and potential publication bias.

For longitudinal evidence, the certainty of the association between obesity and subsequent depression was rated as moderate. Although observational evidence is initially considered low in certainty, the relatively consistent direction and magnitude of effects across studies supported an increased level of confidence. However, the small number of included studies and potential imprecision limited the overall certainty.

For cross-sectional evidence, the certainty was rated as low, reflecting a substantial heterogeneity across studies, a variability in measurement approaches, and the inherent limitations of cross-sectional designs in establishing temporal relationships. These factors led to a reduced confidence in the stability and interpretability of the pooled estimates.

The results of the GRADE assessment are summarized in Table 7.

## 4. Discussion

### 4.1. Summary of Main Findings

In the present study, a stratified meta-analysis examining the association between obesity and depression among children and adolescents was conducted. By separating longitudinal and cross-sectional evidence and restricting the meta-analysis to studies with comparable exposure and outcome definitions, our analysis was able to provide a clearer evaluation of the temporal relationship between obesity and depressive outcomes in youth.

The longitudinal meta-analysis indicated that obesity was associated with an increased likelihood of subsequent depression (OR = 2.05, 95% CI: 1.40–2.99). The observed association was moderate in magnitude and remained consistent across studies, although one of the included longitudinal studies reported a non-significant effect. Across the four prospective cohort studies included in this primary meta-analysis, adolescents with obesity were more likely to develop subsequent depressive disorders compared with their normal-weight peers (Anderson et al., 2007; Boutelle et al., 2010; Marmorstein et al., 2014; Roberts & Duong, 2013). The pooled effect size was relatively consistent across studies, and statistical heterogeneity was minimal. However, given the small number of included studies (k = 4), the observed I^2^ = 0% should be interpreted cautiously, as it may reflect limited statistical power rather than true homogeneity. Sensitivity analyses indicated that the association remained stable across leave-one-out models, suggesting that the overall estimate was not driven by any single study.

In contrast, the cross-sectional meta-analysis examining the concurrent association between obesity and depression produced a weaker and statistically non-significant pooled effect. Although the direction of the association was positive, the magnitude of the relationship was smaller, and the heterogeneity across studies was considerably higher than in the longitudinal analysis. Together, these findings suggest that the temporal relationship between obesity and depression may be more clearly detectable in prospective study designs than in cross-sectional studies.

Taken together, the results indicate that obesity during adolescence may be associated with subsequent depressive outcomes, while the concurrent associations observed in cross-sectional studies appear to be less consistent. These findings highlight the importance of considering study design and temporal ordering when evaluating the relationship between obesity and mental health outcomes during adolescence (Luppino et al., 2010; Mannan et al., 2016). However, given the limited number of longitudinal studies included in the meta-analysis, these findings should be considered preliminary and interpreted with caution.

### 4.2. Interpretation of Longitudinal Evidence

The prospective association observed in the longitudinal analysis is consistent with several theoretical mechanisms linking obesity to later depressive outcomes. One widely discussed explanation involves psychosocial processes related to weight stigma and social evaluation. Adolescence represents a developmental stage characterized by heightened sensitivity to peer feedback and body image concerns, and adolescents with obesity may experience teasing, discrimination, or social marginalization related to body weight (Puhl & Heuer, 2009). These experiences may contribute to negative self-perceptions, reduced self-esteem, and emotional distress, which may in turn increase vulnerability to depressive disorders (Griffiths et al., 2010).

In addition to psychosocial pathways, biological mechanisms have also been proposed to explain the association between obesity and depression. Research has suggested that inflammatory processes associated with excess adiposity may influence the neurobiological systems involved in mood regulation (Milaneschi et al., 2019). Chronic low-grade inflammation, metabolic dysregulation, and alterations in neuroendocrine pathways have all been proposed as potential mechanisms linking obesity with depressive symptomatology.

Behavioral factors may also contribute to the observed association. Adolescents with obesity may be less likely to engage in physical activity and may experience sleep disturbances or other lifestyle factors associated with poorer mental health outcomes (Chaput et al., 2016). Such behavioral patterns may interact with psychosocial and biological processes, creating a complex network of factors that contribute to the development of depressive disorders over time.

Importantly, the relationship between obesity and depression may not be unidirectional. Previous longitudinal research has suggested that depressive symptoms may also increase the risk of subsequent weight gain, indicating that the association between these conditions may operate in both directions (Luppino et al., 2010; Mannan et al., 2016). Depression may influence health behaviors such as physical activity, sleep patterns, and dietary intake, which in turn may contribute to increases in body weight over time. Consequently, the association observed in prospective studies examining obesity as a predictor of depression may represent only one component of a more complex reciprocal relationship between physical and mental health during adolescence.

Although longitudinal designs provide information about temporal ordering, they do not establish causality. Residual confounding and unmeasured variables may influence the observed associations, and therefore causal interpretations should be avoided.

### 4.3. Interpretation of Cross-Sectional Findings

The cross-sectional meta-analysis revealed a weaker and statistically non-significant association between obesity and depression. Several factors may explain this pattern. First, cross-sectional studies capture associations between variables measured at the same time point and therefore cannot establish temporal ordering. As a result, a cross-sectional design cannot determine whether obesity precedes depression, whether depression contributes to weight gain, or whether both conditions arise from shared underlying determinants (Luppino et al., 2010).

Second, the cross-sectional studies examining obesity and depression in adolescents are characterized by a substantial methodological heterogeneity. Studies differ in their definitions of obesity, the instruments used to measure depressive symptoms, and the statistical approaches employed to estimate associations (Mannan et al., 2016). Some studies rely on diagnostic interviews, while others use self-reporting screening instruments that assess depressive symptom severity rather than clinical diagnoses. The substantial heterogeneity observed in the cross-sectional meta-analysis likely reflects both methodological and substantive sources of variation across studies. Differences in age ranges, measurement approaches (e.g., diagnostic interviews versus self-report symptom scales), and operational definitions of obesity and depression may contribute to the variability in effect estimates. In addition, heterogeneity may arise from broader contextual factors, including cultural norms, socioeconomic conditions, and differing levels of weight-related stigma, which may shape the psychological impact of obesity across populations (Mannan et al., 2016).

Importantly, heterogeneity in observational meta-analyses is not merely a statistical artifact but may reflect meaningful differences in underlying mechanisms and population characteristics (Higgins et al., 2003; Borenstein et al., 2009). In the context of the present study, the moderate heterogeneity observed suggests that the association between obesity and depression is not uniform but may depend on specific individual and contextual factors. This variability underscores the need for a cautious interpretation of pooled cross-sectional estimates and highlights the importance of examining potential moderators in future research. This pattern is consistent with prior literature suggesting that associations between obesity and mental health outcomes in youth are context-dependent rather than universal.

Another important consideration concerns the distinction between objectively measured obesity and adolescents’ subjective perceptions of body weight. Several studies have suggested that perceived overweight status or body dissatisfaction may be more strongly associated with depressive symptoms than BMI itself (Needham & Crosnoe, 2005; Wardle et al., 2006). Adolescents who perceive themselves as overweight may experience greater body image concerns, social comparison processes, and fear of stigma, which may increase vulnerability to emotional distress even in the absence of clinical obesity. These findings suggest that psychosocial interpretations of body weight may play an important role in understanding the relationship between obesity and depression during adolescence.

Cultural and social factors may also influence how body weight affects psychological well-being. Differences in body image norms, social expectations, and stigma across populations may shape the psychological consequences of excess body weight, potentially contributing to the heterogeneity observed in cross-sectional findings (Puhl & Heuer, 2009).

### 4.4. Integration with Previous Meta-Analyses

Previous meta-analytic studies have examined the association between obesity and depression across different populations and age groups. For example, Luppino et al. (2010) reported that obesity was associated with an increased risk of subsequent depression, while Mannan et al. (2016) identified modest prospective associations between obesity and depressive outcomes across the course of life.

The findings of the present study are broadly consistent with these earlier meta-analyses but extend the literature in several important ways. In particular, previous meta-analyses have often combined cross-sectional and longitudinal evidence within the same pooled analysis. Although this approach provides an overall summary of the association between obesity and depression, it may obscure important differences arising from different study designs that address distinct research questions (Borenstein et al., 2009).

By separating prospective longitudinal evidence from cross-sectional findings, our present analysis provides a clearer evaluation of the temporal association between obesity and depression. This stratified approach allows for a more precise interpretation of whether obesity may precede the development of depressive disorders, rather than merely co-occurring with depressive symptoms. While the findings of the longitudinal meta-analysis suggest that obesity is associated with an increased risk of subsequent depression, given the limited number of studies and the observational nature of the data, causal inferences should be made with caution.

### 4.5. Study Strengths

The present study has several strengths that should be considered when interpreting the findings. First, our systematic search strategy was comprehensive and followed established guidelines, increasing the likelihood that relevant studies were identified and included (Page et al., 2021). Second, the inclusion criteria were clearly defined, ensuring a consistent selection of studies examining the association between obesity and depression in children and adolescents. Third, the use of established methodological tools, including the Newcastle–Ottawa Scale, allowed for a structured assessment of study quality and risk of bias across the included studies (Wells et al., 2000).

In addition, our use of random-effects meta-analytic models accounted for between-study variability, which is particularly important given the diversity of study populations and measurement approaches in this field (Borenstein et al., 2009). Sensitivity analyses further supported the robustness of the findings, indicating that the overall results were not driven by any single study.

### 4.6. Limitations

Despite these strengths, several limitations should be acknowledged.

First, a key limitation of the present meta-analysis concerns the conceptual heterogeneity of both obesity and depression across studies. This restriction resulted from our intentional methodological choice to prioritize conceptual coherence and the validity of pooled estimates by avoiding the aggregation of non-equivalent constructs, such as continuous symptom scores and clinical diagnoses, even at the cost of reducing the number of eligible studies. Although our quantitative synthesis was restricted to categorical definitions of obesity and binary depression outcomes to improve the comparability of effect sizes, substantial variability remains in the diagnostic criteria, measurement instruments, and operational definitions. This simplification, while methodologically necessary for meta-analytic pooling, may not fully capture the complexity of these constructs and may limit the interpretability and comparability of findings across studies.

Second, the number of studies included in both the longitudinal and cross-sectional meta-analyses was relatively small. Although the pooled estimates were consistent across studies, the limited number of available prospective studies restricts the precision of the pooled estimates and limits our ability to examine potential moderators. Our analysis did not examine potential moderators (e.g., sex, age, or geographic variation), as the small number of included studies precluded reliable subgroup or meta-regression analyses and would likely yield unstable or underpowered estimates. Third, a substantial heterogeneity was observed in the cross-sectional studies, reflecting differences in study design, measurement approaches, and population characteristics (Mannan et al., 2016). Such variability complicates the interpretation of pooled cross-sectional estimates and underscores the need for cautious interpretation.

Fourth, variability in the measurement of both obesity and depression across studies remains an important challenge for meta-analyses. Depression outcomes were assessed using both diagnostic instruments and symptom scales, which capture related but distinct constructs, potentially affecting the comparability of effect estimates. In addition, differences in BMI classification systems, depression assessment tools, and statistical reporting may further limit consistency across studies.

The operational definition of obesity used in the present study—based on BMI percentiles (≥95th percentile)—also has inherent limitations. BMI percentiles are distribution-based and dependent on population norms, meaning that shifts in population weight distributions may influence classification thresholds over time. Moreover, BMI does not distinguish between fat mass and lean mass, which may result in misclassification, particularly among more muscular adolescents. The restriction to English-language studies may introduce language bias and may have led to the exclusion of relevant studies published in other languages. Taken together, these measurement-related limitations may attenuate or inflate the observed associations and should be considered when interpreting the findings.

Fifth, the interpretation of heterogeneity estimates in our longitudinal meta-analysis is limited by the small number of included studies. With a small number of studies, statistical tests for heterogeneity have a low power, and true between-study variability may not be detected. This is reflected in the wide confidence interval for I^2^, indicating a substantial uncertainty in the heterogeneity estimate.

Sixth, although formal statistical tests did not indicate strong evidence of small-study effects, the possibility of publication bias cannot be entirely excluded, particularly given the limited number of studies included in the analyses (Peters et al., 2006).

Furthermore, although the GRADE framework was used to assess the overall certainty of evidence, these ratings are based on structured judgment and may be influenced by the limited number of studies and variability in study design, particularly for cross-sectional evidence.

Finally, the majority of included studies were conducted in the United States, which may limit the generalizability of the findings to other cultural and geographic contexts.

### 4.7. Novel Contributions of the Present Study

In our present study, we contribute to the existing literature by applying a stratified meta-analytic framework that separates longitudinal from cross-sectional evidence, addressing a key limitation of previous meta-analyses that have often combined heterogeneous study designs into a single pooled estimate (e.g., Borenstein et al., 2009). This distinction allows for a more conceptually consistent evaluation of the temporal relationship between obesity and depression.

Importantly, by focusing the longitudinal synthesis on studies with comparable exposure and outcome definitions, our present meta-analysis provides more precise evidence that adolescent obesity is associated with an increased risk of subsequent depression. This contributes to clarifying an area of the literature that has previously yielded mixed and sometimes contradictory findings, particularly due to the variability in measurement approaches and study designs.

From a clinical perspective, these findings suggest that obesity in adolescence may function not only as a concurrent correlate but also as a potential early risk marker for later depressive disorders, highlighting the importance of integrated prevention strategies that address both physical and mental health during adolescence.

From a research perspective, our results emphasize the need for future studies to adopt more consistent operational definitions of obesity and depression, and to prioritize longitudinal designs. Additionally, the observed variability across studies underscores the importance of examining moderators such as sex, socioeconomic status, and cultural context, which remain underexplored in the current literature.

### 4.8. Directions for Future Research

Future research should aim to better understand the variability observed in the association between obesity and depression across studies. The moderate heterogeneity identified in the cross-sectional meta-analysis suggests that this relationship is not uniform, but may depend on specific individual, contextual, and methodological factors (Mannan et al., 2016). Future studies should therefore prioritize identifying conditions under which obesity is more strongly associated with depressive outcomes.

An important direction for future research is the examination of potential bidirectional relationships between obesity and depression. While the present findings support the role of obesity as a predictor of subsequent depression, previous longitudinal evidence has also suggested that depressive symptoms may increase the risk of later obesity (Luppino et al., 2010). Clarifying these reciprocal pathways requires well-powered longitudinal designs with multiple assessment points and consistent measurement strategies.

In addition, future research should more explicitly differentiate between objective measures of obesity and subjective perceptions of body weight. Emerging evidence suggests that perceived overweight and body dissatisfaction may be more strongly associated with depressive symptoms than objective BMI alone (Roberts & Duong, 2013; Sutaria et al., 2019). Studies incorporating measures of weight perception, stigma, and body image are therefore essential for a more comprehensive understanding of the psychological impact of obesity during adolescence.

Further work is also needed to investigate the underlying mechanisms linking obesity and depression. Integrating psychosocial, behavioral, and biological factors—such as weight-related stigma, social isolation, inflammatory processes, and health behaviors—may help clarify the pathways through which these conditions are associated (Milaneschi et al., 2019; Puhl & Heuer, 2009).

Finally, future studies should consider developmental timing and broader contextual influences. Differences across age groups, sex, socioeconomic status, and cultural norms related to body image may shape the strength and direction of the association between obesity and depression. Expanding research beyond predominantly U.S.-based samples will be particularly important for improving the generalizability of findings.

## 5. Conclusions

In conclusion, the findings of this meta-analysis suggest a possible longitudinal association between obesity during childhood and adolescence and an increased likelihood of subsequent depression. Evidence from prospective studies indicates a generally consistent pattern, whereas cross-sectional findings appear more heterogeneous and less robust. However, the strength and causal nature of this relationship remain uncertain, given the observational design and the limited number of longitudinal studies. Continued longitudinal research will be essential for clarifying the mechanisms underlying this association and for informing prevention strategies targeting both physical and mental health outcomes in adolescents.

## Figures and Tables

**Table 1:** Characteristics of studies included in the meta-analytic framework.

Study	Country	Design	Total N	Obesity N	Reference N	Age (Years)	Sex (% Female)	Obesity Definition	Depression Measure	Outcome	Analytical Tier
Sjöberg et al. (2005)	Sweden	Cross-sectional	4703	131	4159	15–17	49.2	IOTF cutoffs	DSRS	Binary depression	Core (included in meta-analysis)
Swallen et al. (2005)	USA	Cross-sectional	4743	292	3189	12–20	51.8	BMI ≥ 97th + 2 units	CES-D	Binary symptoms	Core (included in meta-analysis)
Needham and Crosnoe (2005)	USA	Cross-sectional	18,924	NR	NR	11–21	50.9	BMI continuous	CES-D	Continuous symptoms	Core Extended (not pooled)
Wardle et al. (2006)	UK	Cross-sectional	4320/1824	223/79	3235/1377	11/14–15	40.3/68.4	IOTF cutoffs	SDQ/CES-D	Continuous symptoms	Core Extended (not pooled)
Özmen et al. (2007)	Turkey	Cross-sectional	2101	24	2077	15–18	50.0	IOTF cutoffs	CDI	Continuous symptoms	Core Extended (not pooled)
Anderson et al. (2007)	USA	Longitudinal	701	45	NR	12–17.99	50.2	BMI ≥ 95th (CDC)	DISC/SCID-IV	MDD/anxiety	Core (included in meta-analysis)
Bazargan-Hejazi et al. (2010)	USA	Cross-sectional	3892	465	2814	12–17	49.4	BMI > 95th	CES-D (8-item)	Binary symptoms	Core (included in meta-analysis)
Boutelle et al. (2010)	USA	Longitudinal	496	NR	NR	11–15	100.0	BMI ≥ 95th (CDC)	K-SADS	MDD/symptoms	Core (included in meta-analysis)
Merikangas et al. (2012)	USA	Cross-sectional	4150	NR	NR	12–19	48.3	BMI ≥ 95th (CDC)	DISC-IV	MDD	Core (included in meta-analysis)
Roberts and Duong (2013)	USA	Longitudinal	3134	NR	Healthy weight	11–17	48.9	BMI > 95th	DSM-IV interview	Depression	Core (included in meta-analysis)
Marmorstein et al. (2014)	USA	Longitudinal	1512	NR	NR	11, 14, 17, 20, 24	50.3	BMI ≥ 95th (<20); ≥30 (≥20)	DISC/SCID	MDD	Core (included in meta-analysis)
Hammerton et al. (2014)	UK	Longitudinal	289/614/4861	NR	NR	9–19	57.1/56.8/52.1	IOTF categories	CAPA/DAWBA	Binary + continuous	Core Extended (not pooled)
Wang et al. (2014)	Hong Kong	Longitudinal	5797	NR	NR	12–15	48.2	BMI z-score	PHQ-9	Continuous symptoms	Core Extended (not pooled)
Pryor et al. (2016)	Canada	Longitudinal	1221	134–203	884	6–13	54.0	IOTF cutoffs	CDI	Internalizing symptoms	Core Extended (not pooled)
Delgado-Floody et al. (2020)	Chile	Cross-sectional	598	NR	NR	10–13	45.0	BMI ≥ 95th (CDC)	CDI	Depressive symptoms	Core (included in meta-analysis)
Moonajilin and Gozal (2020)	Bangladesh	Cross-sectional	622	81	541	13–18	43.6	BMI ≥ 85th (OW/OB)	PHQ-9	Binary depression/anxiety	Core Extended (not pooled)
Smith and Mason (2022)	USA	Cross-sectional	11,708	1938	7793	9–10	52.1	BMI-z > 1.64	K-SADS	MDD	Core (included in meta-analysis)
Colozza et al. (2025)	Indonesia	Longitudinal	29,029	282/10,214	3078/15,455	14–19/≥20	53.5/48.2	BMI ≥ 23/age-specific	CES-D-10	Continuous symptoms	Core Extended (not pooled)

Note. Core = studies included in the quantitative meta-analysis; Core Extended = studies retained as supplementary evidence but not entered into pooled analyses because of differences in design, exposure, or outcome measurement. BMI = body mass index; CDC = Centers for Disease Control and Prevention; IOTF = International Obesity Task Force; CES-D = Center for Epidemiologic Studies Depression Scale; DSRS = Depression Self-Rating Scale; CDI = Children’s Depression Inventory; PHQ-9 = Patient Health Questionnaire-9; K-SADS = Kiddie Schedule for Affective Disorders and Schizophrenia; DISC = Diagnostic Interview Schedule for Children; SCID = Structured Clinical Interview for DSM Disorders; CAPA = Child and Adolescent Psychiatric Assessment; DAWBA = Development and Well-Being Assessment; MDD = major depressive disorder; NR = not reported; OW/OB = overweight/obese. Where only prevalence rates were available, absolute counts were not derived to avoid introducing estimation error.

**Table 2:** Longitudinal studies included in the core meta-analysis examining obesity as a predictor of subsequent depression.

Study	Country	Sample Size	Age at Baseline	Obesity Definition	Depression Outcome	Effect Estimate	95% CI	Adjusted (Age/Sex)
Anderson et al. (2007)	USA	701	12–17.99 years	BMI ≥ 95th percentile	Major depressive disorder (DSM-IV)	HR = 2.10	1.01–4.36	Yes
Boutelle et al. (2010)	USA	496	11–15 years	BMI ≥ 95th percentile	Major depression (DSM-IV)	OR = 1.57	0.77–3.38	Yes
Roberts and Duong (2013)	USA	3134	11–17 years	BMI ≥ 95th percentile	Major depression (DSM-IV)	OR = 1.90	0.85–4.25	Yes
Marmorstein et al. (2014)	USA	731	11–24 years	BMI ≥ 95th percentile (<20); BMI ≥ 30 (≥20)	Major depressive disorder (DSM-based)	OR = 2.83	1.32–6.09	Yes

Note. BMI = body mass index; HR = hazard ratio; OR = odds ratio; CI = confidence interval.

**Table 3:** Cross-sectional studies included in the meta-analysis examining the concurrent association between obesity and depression.

Study	Country	Sample Size	Age	Obesity Definition	Depression Measure	Effect Estimate	95% CI
Swallen et al. (2005)	USA	4743	12–20 years	BMI ≥ 97th percentile	CES-D	OR = 0.99	0.60–1.62
Sjöberg et al. (2005)	Sweden	~2500	15–17 years	BMI category (IOTF)	DSRS	OR = 1.67	1.12–2.49
Bazargan-Hejazi et al. (2010)	USA	1830	12–17 years	BMI category	CES-D (8-item)	OR = 0.71	0.42–1.19
Merikangas et al. (2012)	USA	10,123	12–19 years	BMI category	DISC-IV	OR = 1.60	0.90–2.90
Delgado-Floody et al. (2020)	Chile	632	10–13 years	BMI ≥ 95th percentile	CDI	OR = 2.05	1.09–3.88
Smith and Mason (2022)	USA	~450	9–10 years	BMI category	K-SADS	OR = 2.48	1.13–5.44

Note. BMI = body mass index; OR = odds ratio; CI = confidence interval; CES-D = Center for Epidemiologic Studies Depression Scale; DSRS = Depression Self-Rating Scale; CDI = Children’s Depression Inventory; DISC-IV = Diagnostic Interview Schedule for Children; K-SADS = Kiddie Schedule for Affective Disorders and Schizophrenia.

**Table 4:** Sensitivity analysis (leave-one-out) for the cross-sectional meta-analysis.

Omitted Study	Pooled OR	95% CI	I^2^
Swallen et al. (2005)	1.52	1.00–2.30	62.70%
Sjöberg et al. (2005)	1.35	0.86–2.11	64.65%
Bazargan-Hejazi et al. (2010)	1.59	1.19–2.11	25.69%
Merikangas et al. (2012)	1.37	0.89–2.11	67.67%
Delgado-Floody et al. (2020)	1.31	0.88–1.95	63.34%
Smith and Mason (2022)	1.29	0.89–1.88	61.15%

Note: OR = odds ratio; CI = confidence interval.

**Table 6:** Risk-of-bias assessment by domain for included studies.

Study	Selection Bias	Comparability (Confounding)	Exposure Measurement	Outcome Measurement	Follow-Up (Longitudinal Only)	Overall Risk
Anderson et al. (2007)	Low	Moderate	Low	Low	Low	Low
Boutelle et al. (2010)	Low	Moderate	Low	Low	Low	Low
Roberts and Duong (2013)	Low	Moderate	Low	Low	Low	Low
Marmorstein et al. (2014)	Low	Moderate	Low	Low	Low	Low
Swallen et al. (2005)	Low	High	Moderate	Moderate	N/A	Moderate
Sjöberg et al. (2005)	Low	High	Moderate	Moderate	N/A	Moderate
Bazargan-Hejazi et al. (2010)	Moderate	High	Moderate	Moderate	N/A	High
Merikangas et al. (2012)	Low	Moderate	Moderate	Moderate	N/A	Moderate
Delgado-Floody et al. (2020)	Moderate	High	Moderate	Moderate	N/A	High
Smith and Mason (2022)	Moderate	High	Moderate	Moderate	N/A	High

Note. Risk of bias was assessed using a domain-based framework focusing on selection bias, comparability (control of confounding), exposure and outcome measurement, and follow-up (for longitudinal studies). Overall risk was determined based on consistency across domains.

**Table 7:** Certainty of evidence (GRADE).

Outcome	Study Design	Risk of Bias	Inconsistency	Indirectness	Imprecision	Publication Bias	Certainty
Obesity → Depression (Longitudinal)	Observational	Not serious	Not serious	Not serious	Serious	Undetected	Moderate
Obesity ↔ Depression (Cross-sectional)	Observational	Serious	Serious	Not serious	Serious	Possible	Low

Note. Certainty of evidence was assessed using the GRADE framework. Observational evidence was initially rated as low in certainty and subsequently upgraded or downgraded based on the risk of bias, inconsistency, indirectness, imprecision, and publication bias. Longitudinal evidence was rated as moderate in certainty due to consistent findings but limited by imprecision, whereas cross-sectional evidence was rated as low in certainty due to heterogeneity, methodological variability, and inherent design limitations.

## Data Availability

The original contributions presented in this study are included in the article/Supplementary Materials. Further inquiries can be directed to the corresponding authors.

## Supplementary Materials

The following supporting information can be downloaded at: https://www.mdpi.com/article/10.3390/bs16050671/s1.
